# Clinical and Functional Outcomes of Intra-articular Hyaluronic Acid Versus Corticosteroids in Knee Osteoarthritis: A Comparative Study

**DOI:** 10.7759/cureus.84712

**Published:** 2025-05-23

**Authors:** Richik Sarkar, K Vinod Kumar, JS Nagakumar, Arun Kumaar

**Affiliations:** 1 Department of Orthopaedics, Sri Devaraj Urs Academy of Higher Education and Research, Kolar, IND

**Keywords:** corticosteroids, functional improvement, hyaluronic acid, intra-articular injection, knee osteoarthritis, pain management

## Abstract

Background: Knee osteoarthritis (OA) ranks among the top causes of pain and disability globally, with intra-articular hyaluronic acid (HA) and corticosteroids (CS) frequently employed for symptom relief. HA enhances joint lubrication and has anti-inflammatory properties, while CS provides rapid pain relief through inflammation suppression. This study evaluates the effectiveness of HA and CS injections in patients with Grade II and III knee OA.

Methods: A cross-sectional study was carried out involving 60 patients with Grade II or III knee OA (ages 40-60). The subjects were randomised to receive either intra-articular CS (40 mg triamcinolone acetonide with 2% lignocaine) or HA (6 ml, 48 mg sodium hyaluronate). At baseline, two weeks, six weeks, and three months after treatment, a numerical rating scale (NRS) and the Western Ontario and McMaster Universities Osteoarthritis Index (WOMAC) were used to measure pain and functional outcomes, respectively. The unpaired t-test and repeated measures ANOVA were among the statistical analyses used.

Results: HA and CS groups significantly improved NRS and WOMAC scores over time (p = 0.001). However, the HA group exhibited superior pain reduction and functional improvement from six weeks onward. At three months, the median NRS score was significantly lower in the HA group (2 (IQR 1-2)) compared to the CS group (3 (IQR 3-4), p = 0.001). Similarly, the HA group's WOMAC score was significantly better (53.4 ± 9.91 vs. 64.2 ± 6.8, p = 0.001).

Conclusion: While both HA and CS injections effectively reduce pain and improve functionality of the knee joint, HA provides sustained pain relief and functional improvement beyond the short-term effects of CS. These findings support the use of HA as a preferred option for managing mild-to-moderate knee OA.

## Introduction

Osteoarthritis (OA) is the primary cause of knee pain and the predominant reason for disability globally [1]. OA is a long-term, progressive joint disease characterised by secondary hyperosteogeny and articular cartilage degradation. This condition causes significant knee joint pain and affects 35% of adults over 65 [2]. The quality of life of the patient with OA is directly affected, in addition to the physical and/or mental comorbidity, stiffness, decreased mobility, and chronic pain. Owing to its incredible complexity and the interaction of many biological factors, including ageing, sex hormone deficiency, and genetic alterations, OA is not well understood [3]. Recent research has concentrated on molecular indicators connected to stress-induced chondrocyte senescence. OA is caused by several mechanical and metabolic processes that break down cartilage. Most knee OA research has focused on tertiary management options, like pain reduction with efficient medication therapies (NSAIDs, chondroitin supplements, etc.). Although long-term use of NSAIDs has been associated with potentially dangerous side effects, they do help treat OA pain [4]. In addition, their customized response varies greatly due to pharmacogenomics associations. There is no known cure for OA except for knee joint replacement, which is typically reserved for end-stage cases.

In cartilage and synovial fluid, HA is a widely distributed, high molecular weight material. It serves a number of purposes in the joint, such as lubricating, filling in gaps to maintain joint mobility, and controlling cellular processes like protein binding [5]. The endogenous HA in the joint breaks down from a high molecular weight (6500-10,900 kDa) to a lower molecular weight (2700-4500 kDa) as OA worsens [6]. As a result, the affected joint's synovial fluid has less mechanical and viscoelastic qualities. It has been demonstrated that intra-articular HA injections enhance synovial fluid viscosity and joint lubrication, restore hyaluronan synthesis, prevent proteoglycan degradation, and provide analgesic and anti-inflammatory benefits when treating OA. [6] Intra-articular corticosteroids exert anti-inflammatory effects by blocking the production of inflammatory cytokines. However, their duration of effect is significantly shorter than the recommended interval between administration doses [7]. Thus, while the short-term effects are satisfactory, additional research is required to determine the long-term effects. This study evaluates the effectiveness of intra-articular hyaluronic acid (HA) versus intra-articular corticosteroid (CS) injections in patients with Grade II and Grade III knee OA, as classified by the Kellgren-Lawrence system.

## Materials and methods

A prospective study was conducted at Sri Devaraj Urs Academy of Higher Education, a tertiary care hospital and medical college in Kolar, India between June 2024 and December 2024, involving 60 patients diagnosed with symptomatic primary OA of the unilateral or bilateral knee joint, characterized by pain lasting more than three months and not responding to oral medications and physiotherapy. Eligible participants were between 40 and 60 years of age, of either gender, with radiologically confirmed Kellgren and Lawrence Grade II or III OA. Patients with secondary pathological conditions such as rheumatoid arthritis or pseudogout, prior history of knee arthroplasty, previous intra-articular injections in the knee, bleeding disorders, local skin infection or pathology, or immunosuppression were excluded from the study. Patients were selected using simple random sampling. Each day, a list of eligible patients fulfilling the criteria was prepared, and a unique number was assigned to each individual. Patients were then randomly selected using a random number table or computer-generated randomization. This ensured each eligible patient had an equal and independent chance of inclusion, reducing selection bias and enhancing representativeness. Recruitment continued daily until the desired sample size of 60 patients was achieved.

After obtaining informed consent, baseline demographic and clinical data were collected. Patients were then randomly allocated into two groups: (1) Group A (HA group) received 6 ml (48 mg) of high molecular weight sodium hyaluronate intra-articularly; (2) Group B (CS group) received 1 ml (40 mg) of triamcinolone acetonide combined with 1 ml of 2% lignocaine intra-articularly.

All injections were administered under strict aseptic precautions using the no-touch needle technique via the anterolateral portal, with the patient in a supine position and the knee flexed to 90 degrees. Synovial fluid was aspirated before injection to confirm intra-articular placement. All procedures were performed by the unit's chief orthopedic consultant to minimize operator variability.

Outcome measures included pain and functional assessment using the Numerical Rating Scale (NRS) and the Western Ontario and McMaster Universities Osteoarthritis Index (WOMAC). These assessments were conducted at baseline, two weeks, six weeks, and six months after the procedure. Follow-up evaluations were carried out by the primary investigator to maintain consistency.

Ethics statement

Ethical clearance was received from the Institutional Ethical Committee of Sri Devaraj Urs Medical College (approval number SDUAHER/KLR/R&D/CEC/S/PG/128/2024-25 to conduct the research.

Sample size

The sample size for the current research was based on a study by Davelillo et al. (2022) [8] where 200 patients were randomized into two groups, each receiving either intra-articular hyaluronic acid (HA) or bone marrow aspirate (BM). Functional improvement was assessed using the WOMAC (Western Ontario and McMaster Universities Osteoarthritis Index) score. At the 12-month follow-up, the HA group demonstrated a 47.95% improvement in WOMAC scores, significantly higher than the 13.2% improvement observed in the BM group [8]. 

With the above study as a reference, the current study employed a two-sided significance level (alpha) of 0.05, allowing for a 7% chance of a Type I error. It was designed with 80% power (beta = 0.2) to detect a significant difference between groups if present. These parameters indicated a required sample size of 30 participants per group, totaling 60 subjects, for adequate statistical power.

Statistical analysis

Variables were analyzed using the Statistical Package for the Social Sciences (SPSS version 30, IBM Corp., Armonk, NY) software after the data were entered into a Microsoft Excel spreadsheet (Microsoft Corp., Redmond, WA). Standard deviation (SD) ± mean was employed to represent continuous variables. A unpaired Student's t-test was utilized for normally distributed variables to compare groups.

## Results

The study enrolled 60 patients, split evenly into hyaluronic acid (HA) and corticosteroid (CS) groups (Table 1). All patients had symptoms for more than three months, and no pre-procedure analgesia was given. Most participants were aged 51-60 years (80% HA, 86.7% CS), with no significant age difference (p = 0.49, Table 1). Females predominated (76.7% HA, 56.7% CS; p = 0.11), and diabetes was the most common comorbidity (76.7% HA, 66.7% CS; p = 0.66, Table 1). Baseline OA severity was similar, with Grade II OA in 83.3% of HA and 90% of CS patients (p = 0.45, Table 2). Initial pain levels, assessed by NRS, showed 93.3% of both groups at a score of 5 (p = 1.00, Table 3).

**Table 1:** Patient demographics and comorbidities Chi-square test for trends. P-value <0.05 is considered significant.

Characteristic	HA Group; n(%)	TA Group; n(%)	Chi-square test for trends value	P-value
Age Group (years)				
41-50	6 (20.0%)	4 (13.3%)	0.80	0.49
51-60	24 (80.0%)	26 (86.7%)		
Gender				
Female	23 (76.7%)	17 (56.7%)	2.7	0.11
Male	7 (23.3%)	13 (43.3%)		
Comorbidities				
DM	23 (76.7%)	20 (66.7%)	0.82	0.66
HTN/DM	3 (10.0%)	5 (16.7%)		
NIL	4 (13.3%)	5 (16.7%)		

**Table 2:** Grading of osteoarthritis Chi-square test. p value <0.05 is considered as significant HA: hyaluronic acid; TA triamcinolone acetonide

Grading of OA	HA group; n (%)	TA group; n (%)	chi-square test	p-value
Grade II	25 (83.3%)	27 (90.0%)	0.57	0.45
Grade III	5 (16.7%)	3 (10.0%)		

**Table 3:** Baseline NRS Score Chi-square test, p-value <0.05 is considered as significant HA: hyaluronic acid; TA triamcinolone acetonide; NRS: numerical rating scale

Baseline NRS Score	HA group; n (%)	TA group; n (%)	chi square test value	p-value
Score 5	28 (93.3%)	28 (93.3%)	0.00	1.00
Score 6	2 (6.7%)	2 (6.7%)		

Pain scores improved significantly over time in both groups as per the Friedman test (HA: χ² = 84.35; CS: χ² = 76.95). However, the HA group consistently demonstrated lower median NRS scores at each follow-up point compared to the CS group, with statistically significant differences observed at two weeks, six weeks, and three months (p = 0.001 for all, Table 4), indicating superior efficacy of hyaluronic acid in reducing pain. Functionally, baseline WOMAC scores were comparable (71.73 HA vs. 70.13 CS; p = 0.51, Table 5). At six weeks, HA scores dropped to 58.4 vs. 65.7 for CS (p = 0.002, Table 5). By three months, HA reached 53.4 vs. 64.2 for CS (p = 0.001, Table 5). Repeated measures ANOVA showed significant functional gains in both groups (p = 0.001, Table 5). Overall, HA provided superior pain relief and functional improvement throughout the study.

**Table 4:** NRS Score over Time Between-group comparisons were performed using the Mann-Whitney test, while within-group changes over time were assessed using the Friedman test. A p-value <0.05 was considered statistically significant. HA: hyaluronic acid; TA triamcinolone acetonide

Time point	HA group (median, IQR)	TA group (median, IQR)	Mann-Whitney test (f value)	p-value
NRS baseline	5 (5-5)	5 (5-5)	450	1.00
NRS 2 weeks	3 (3-4)	4 (4-4)	198	0.001
NRS 6 weeks	2.5 (2-3)	4 (3-4)	112.5	0.001
NRS 3 months	2 (1-2)	3 (3-4)	73	0.001
Friedman Test	84.35	76.95	-	-
P value	0.001	0.001	-	-

**Table 5:** WOMAC Scores over Time Between-group differences were analyzed using an independent t-test, while within-group changes were assessed using repeated measures ANOVA. A p-value <0.05 was considered statistically significant. HA: hyaluronic acid; TA triamcinolone acetonide

Time point	HA group (Mean ± SD)	TA group (Mean ± SD)	Student t-test value	P value
Baseline	71.73 ± 10.57	70.13 ± 7.73	0.67	0.51
2 Weeks	64.67 ± 10.23	67.97 ± 7.3	-1.43	0.16
6 Weeks	58.4 ± 10.2	65.7 ± 7.18	-3.2	0.002
3rd Month	53.4 ± 9.91	64.2 ± 6.8	-4.9	0.001
Repeated Measures ANOVA	154.76	32.48	-	-
P value	0.001	0.001	-	-

## Discussion

This prospective study compared the efficacy of intra-articular hyaluronic acid (HA) and corticosteroid (CS) injections in patients with symptomatic knee OA. Both treatment groups were demographically similar in terms of age, gender distribution, and comorbidities, ensuring a balanced comparison. Our findings demonstrated that while both HA and CS led to significant improvements in pain and functional scores over time, HA consistently outperformed CS at all follow-up points. Notably, patients receiving HA showed greater reductions in pain (NRS) and functional limitations (WOMAC) at two weeks, six weeks, and three months, with statistically significant differences between the groups. These results highlight the superior and sustained therapeutic benefits of HA over CS for knee OA management.

To our knowledge, this is one of the few studies in our regional setting to directly compare HA and CS using a structured follow-up and validated scoring tools. Furthermore, the use of simple random sampling, standardized injection technique by a single senior consultant, and real-world patient profiles, including diabetics, enhances the external validity of our findings. This study contributes to the growing body of evidence suggesting that HA may be preferable for longer-term symptom control in knee OA, particularly in populations with comorbidities where long-term steroid use may pose risks.

He and colleagues highlighted that the prevalence of knee OA increases significantly with age, especially after the age of 50 [9]. Similarly, Delbarre et al. noted that 56% of their knee OA cohort was under 65 years, while Losina et al. estimated the median age of knee OA diagnosis at 55 years [10,11]. By focusing on this age group, our study effectively captured the population most affected by knee OA, ensuring the relevance of evaluating treatment effects in this demographic.

Regarding gender distribution, our study observed a female predominance in both groups, with 76.7% of participants in the HA group and 56.7% in the CS group being women. This trend reflects the global higher prevalence of OA in women. Delbarre et al. reported that 67% of their knee OA cohort was female, while Swain et al. found that globally, women account for 60% of OA cases, with the difference becoming more pronounced after 40 years [10-12]. The Osteoarthritis Action Alliance similarly reported that 62% of individuals with OA are women [13]. Although the gender distribution in our HA group aligns with these findings, the slightly lower percentage of females in the CS group may indicate population-specific variations or random variability within the sample.

Our study revealed diabetes mellitus (DM) as the most common comorbidity, affecting 76.7% of participants in the HA group and 66.7% in the CS group. This prevalence is notably higher than reported in other studies. For example, Swain et al. identified depression, COPD, and hypertension as the most common comorbidities in OA patients after age standardization [12]. The Osteoarthritis Action Alliance reported that 43% of adults with arthritis have diabetes [13]. The high prevalence of DM in our study may reflect specific population characteristics, such as a higher baseline prevalence of diabetes in the region, or selection criteria that favored individuals with metabolic comorbidities.

Regarding the grading of OA, the majority of patients in our study had Grade II OA (83.3% in the HA group and 90.0% in the CS group), with a smaller proportion having Grade III OA. There was no notable difference between the groups (p = 0.45). Our grading system aligns with the widely used Kellgren-Lawrence (KL) scale, which defines Grade II OA as "definite osteophytes and possible narrowing of joint space." Kohn et al. noted this scale as the most commonly employed system in OA research [14]. The predominance of Grade II OA in our study is consistent with trends reported in other research, though some larger studies, such as that by Chen et al., reported a more even distribution across KL grades [15]. The absence of Grade IV OA in our study reflects the focus on mild-to-moderate cases, ensuring the applicability of intra-articular therapies for early-stage disease.

At baseline, most patients in both groups (93.3%) had an NRS (numerical rating scale) score of 5, with a small proportion having a score of 6. This uniformity ensured comparability between groups, as evidenced by the non-significant p-value (p = 1.00). Compared to other studies, our baseline NRS scores are relatively high. For instance, Bannuru et al. reported lower mean baseline NRS scores in their meta-analysis of OA treatments [16]. Additionally, Bellamy et al. observed a wider range of baseline pain scores in their review of OA clinical trials [17]. The high baseline scores in our study reflect a population with significant symptomatic burden, which may influence the magnitude of observed treatment effects.

Both treatment groups experienced significant pain reduction over time, but the HA group demonstrated superior outcomes at all follow-ups. At two weeks, the median NRS score in the HA group was 3 (IQR 3-4), compared to 4 (IQR 4-4) in the CS group (p = 0.001). This difference became more pronounced at six weeks and three months. These findings align with Bannuru et al., who reported greater efficacy of hyaluronic acid beyond eight weeks compared to corticosteroids, and Wang et al., who observed significant pain reduction with hyaluronic acid at three and six months [16, 18]. However, our results differ from those of Leopold et al., who found no significant differences between treatments. Our study's consistent superiority of hyaluronic acid suggests its potential for sustained pain management [19].

As measured by WOMAC scores, functional improvement also showed greater benefits in the Hylasto S group. At six weeks, the mean WOMAC score in the HA group was 58.4 ± 10.2, compared to 65.7 ± 7.18 in the CS group (p = 0.002). By three months, the HA group further improved to 53.4 ± 9.91, compared to 64.2 ± 6.8 in the CS group (p = 0.001). These findings align with studies such as Bellamy et al., who concluded that hyaluronic acid is most effective between 5- and 13-weeks post-injection, and Wang et al., who observed marked reductions in WOMAC scores with hyaluronic acid over six months [17,18]. In contrast, Leopold et al. reported similar functional improvements for both treatments, suggesting that our study population may have experienced unique benefits from hyaluronic acid [19].

Limitations

This study has a few limitations, including the absence of blinding, which introduces potential bias in subjective outcome measures like NRS and WOMAC scores. The diagnosis of OA was made on a radiological basis, and no inflammatory markers were used for the same. Being a single-center study, external validity is restricted. Additionally, the lack of advanced imaging limits objective assessment of structural changes.

## Conclusions

Our study indicates that hyaluronic acid and corticosteroids effectively reduced pain and improved the functionality of the knee joint. However, hyaluronic acid offers significant pain relief and functional improvement, particularly in the short term. The unique characteristics of our study population, such as a high prevalence of diabetes and consistent baseline pain scores, may have contributed to these findings. These findings support the use of hyaluronic acid as the preferred treatment for long-term mild-to-moderate knee OA.

## References

[1] Neogi T (2013). The epidemiology and impact of pain in osteoarthritis. Osteoarthritis Cartilage.

[2] Nikam MA, Patil PV, Kumar P (2022). Effectiveness of intra-articular hyaluronic acid versus corticosteroids in knee osteoarthritis: a comparative study. Int J Res Orthop.

[3] Wojcieszek A, Kurowska A, Majda A, Liszka H, Gądek A (2022). The impact of chronic pain, stiffness and difficulties in performing daily activities on the quality of life of older patients with knee osteoarthritis. Int J Environ Res Public Health.

[4] InformedHealth.org InformedHealth.org (2024). Osteoarthritis of the knee: Learn More - Which painkillers help in osteoarthritis of the knee?. Health Care (IQWiG.

[5] Altman RD, Manjoo A, Fierlinger A, Niazi F, Nicholls M (2015). The mechanism of action for hyaluronic acid treatment in the osteoarthritic knee: a systematic review. BMC Musculoskelet Disord.

[6] Chavda S, Rabbani SA, Wadhwa T (2022). Role and effectiveness of intra-articular injection of hyaluronic acid in the treatment of knee osteoarthritis: a systematic review. Cureus.

[7] Ma L, Zheng X, Lin R (2022). Knee osteoarthritis therapy: Recent advances in intra-articular drug delivery systems. Drug Des Devel Ther.

[8] Trueba Davalillo CÁ, Trueba Vasavilbaso C, Navarrete Álvarez JM, Coronel Granado P, García Jiménez OA, Gimeno Del Sol M, Gil Orbezo F (2015). Clinical efficacy of intra-articular injections in knee osteoarthritis: a prospective randomized study comparing hyaluronic acid and betamethasone. Open Access Rheumatol.

[9] He WW, Kuang MJ, Zhao J (2017). Efficacy and safety of intraarticular hyaluronic acid and corticosteroid for knee osteoarthritis: A meta-analysis. Int J Surg.

[10] Delbarre A, Amor B, Bardoulat I, Tetafort A, Pelletier-Fleury N (2017). Do intra-articular hyaluronic acid injections delay total knee replacement in patients with osteoarthritis - A Cox model analysis. PLoS One.

[11] Losina E, Weinstein AM, Reichmann WM (2013). Lifetime risk and age at diagnosis of symptomatic knee osteoarthritis in the US. Arthritis Care Res (Hoboken).

[12] Swain S, Sarmanova A, Coupland C, Doherty M, Zhang W (2020). Comorbidities in osteoarthritis: A systematic review and meta-analysis of observational studies. Arthritis Care Res (Hoboken).

[13] (2025). Osteoarthritis Action Alliance. OA Prevalence and Burden. https://oaaction.unc.edu/oa-module/oa-prevalence-and-burden/.

[14] Kohn MD, Sassoon AA, Fernando ND (2016). Classifications in brief: Kellgren-Lawrence classification of osteoarthritis. Clin Orthop Relat Res.

[15] Chen P, Gao L, Shi X, Allen K, Yang L (2019). Fully automatic knee osteoarthritis severity grading using deep neural networks with a novel ordinal loss. Comput Med Imaging Graph.

[16] Bannuru RR, Natov NS, Obadan IE, Price LL, Schmid CH, McAlindon TE (2009). Therapeutic trajectory of hyaluronic acid versus corticosteroids in the treatment of knee osteoarthritis: a systematic review and meta-analysis. Arthritis Rheum.

[17] Bellamy N, Campbell J, Robinson V, Gee T, Bourne R, Wells G (2005). Viscosupplementation for the treatment of osteoarthritis of the knee. Cochrane Database Syst Rev.

[18] Wang CT, Lin J, Chang CJ (2004). Therapeutic effects of hyaluronic acid on osteoarthritis of the knee. A meta-analysis of randomized controlled trials. J Bone Joint Surg Am.

[19] Leopold SS, Redd BB, Warme WJ, Wehrle PA, Pettis PD, Shott S (2003). Corticosteroid compared with hyaluronic acid injections for the treatment of osteoarthritis of the knee. A prospective, randomized trial. J Bone Joint Surg Am.

